# 2-Anilino-4-(1,3-benzothia­zol-2-yl)-5-(4-chloro­benzo­yl)thio­phene-3-carbonitrile

**DOI:** 10.1107/S1600536812032588

**Published:** 2012-07-25

**Authors:** Hoong-Kun Fun, Tze Shyang Chia, Hatem A. Abdel-Aziz

**Affiliations:** a X-ray Crystallography Unit, School of Physics, Universiti Sains Malaysia, 11800 USM, Penang, Malaysia; b Department of Pharmaceutical Chemistry, College of Pharmacy, King Saud University, PO Box 2457, Riyadh 11451, Saudi Arabia

## Abstract

In the title compound, C_25_H_14_ClN_3_OS_2_, the central thio­phene ring [maximum deviation = 0.011 (1) Å] makes dihedral angles of 55.72 (5), 13.36 (5) and 46.77 (4)° with the adjacent chloro-substituted benzene ring, the benzene ring and the 1,3-benzothia­zole ring system [maximum deviation = 0.012 (1) Å], respectively. An intra­molecular C—H⋯S(thienyl) hydrogen bond generates an *S*(6) ring motif in the mol­ecule. In the crystal, mol­ecules are linked by pairs of N—H⋯N hydrogen bonds into inversion dimers and the dimers are further connected by C—H⋯O hydrogen bonds into tapes running along [100]. Aromatic π–π stacking inter­actions are also observed [centroid-to-centroid distances = 3.6116 (6) and 3.7081 (6) Å].

## Related literature

For background to the chemistry and biological activity of thio­phenes, see: Fun *et al.* (2012 ▶); Abdel-Aziz *et al.* (2012 ▶). For hydrogen-bond motifs, see: Bernstein *et al.* (1995 ▶). For the stability of the temperature controller used for the data collection, see: Cosier & Glazer (1986 ▶).
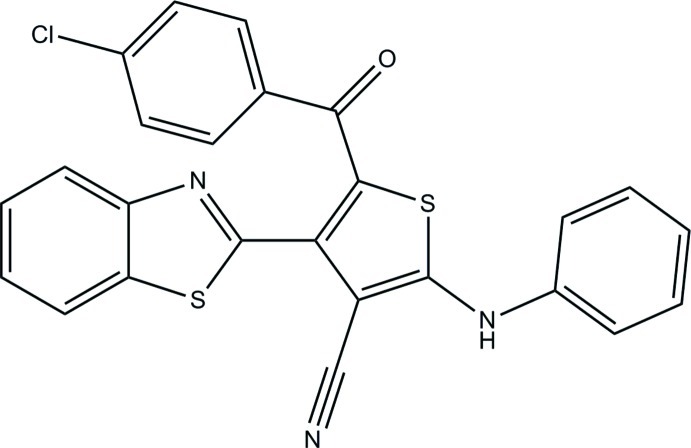


## Experimental

### 

#### Crystal data

C_25_H_14_ClN_3_OS_2_
*M* *_r_* = 471.96
Triclinic, 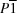

*a* = 6.9469 (2) Å
*b* = 7.6722 (3) Å
*c* = 20.9874 (7) Å
α = 91.519 (1)°
β = 97.577 (1)°
γ = 107.791 (1)°
*V* = 1053.15 (6) Å^3^
*Z* = 2
Mo *K*α radiation
μ = 0.40 mm^−1^
*T* = 100 K
0.31 × 0.27 × 0.19 mm

#### Data collection

Bruker APEX DUO CCD diffractometer
Absorption correction: multi-scan (*SADABS*; Bruker, 2009 ▶) *T*_min_ = 0.884, *T*_max_ = 0.929
27413 measured reflections
7637 independent reflections
6820 reflections with *I* > 2σ(*I*)
*R*_int_ = 0.020

#### Refinement

*R*[*F* ^2^ > 2σ(*F* ^2^)] = 0.029
*wR*(*F* ^2^) = 0.083
*S* = 1.04
7637 reflections
293 parameters
H atoms treated by a mixture of independent and constrained refinement
Δρ_max_ = 0.48 e Å^−3^
Δρ_min_ = −0.20 e Å^−3^

### 

Data collection: *APEX2* (Bruker, 2009 ▶); cell refinement: *SAINT* (Bruker, 2009 ▶); data reduction: *SAINT*; program(s) used to solve structure: *SHELXTL* (Sheldrick, 2008 ▶); program(s) used to refine structure: *SHELXTL*; molecular graphics: *SHELXTL*; software used to prepare material for publication: *SHELXTL* and *PLATON* (Spek, 2009 ▶).

## Supplementary Material

Crystal structure: contains datablock(s) global, I. DOI: 10.1107/S1600536812032588/hb6899sup1.cif

Structure factors: contains datablock(s) I. DOI: 10.1107/S1600536812032588/hb6899Isup2.hkl

Supplementary material file. DOI: 10.1107/S1600536812032588/hb6899Isup3.cml

Additional supplementary materials:  crystallographic information; 3D view; checkCIF report

## Figures and Tables

**Table 1:** Hydrogen-bond geometry (Å, °)

*D*—H⋯*A*	*D*—H	H⋯*A*	*D*⋯*A*	*D*—H⋯*A*
N3—H1N3⋯N2^i^	0.862 (15)	2.163 (15)	2.9558 (12)	152.7 (13)
C17—H17*A*⋯O1^ii^	0.95	2.60	3.3496 (14)	136
C24—H24*A*⋯S2	0.95	2.49	3.1719 (9)	128

Symmetry codes: (i) 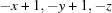; (ii) 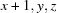.

**Fig. 1.**
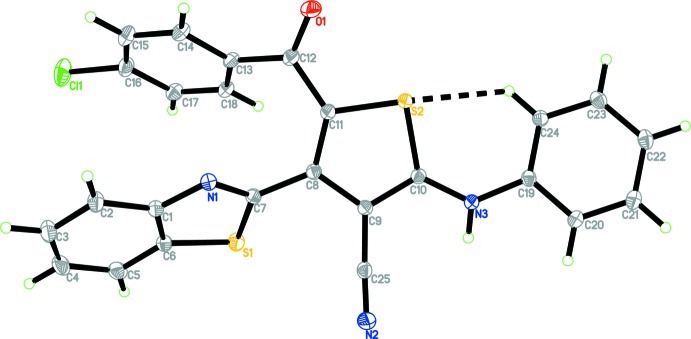
The molecular structure of the title compound with 50% probability displacement ellipsoids. The dashed line represents the intramolecular C—H···S hydrogen bond.

**Fig. 2.**
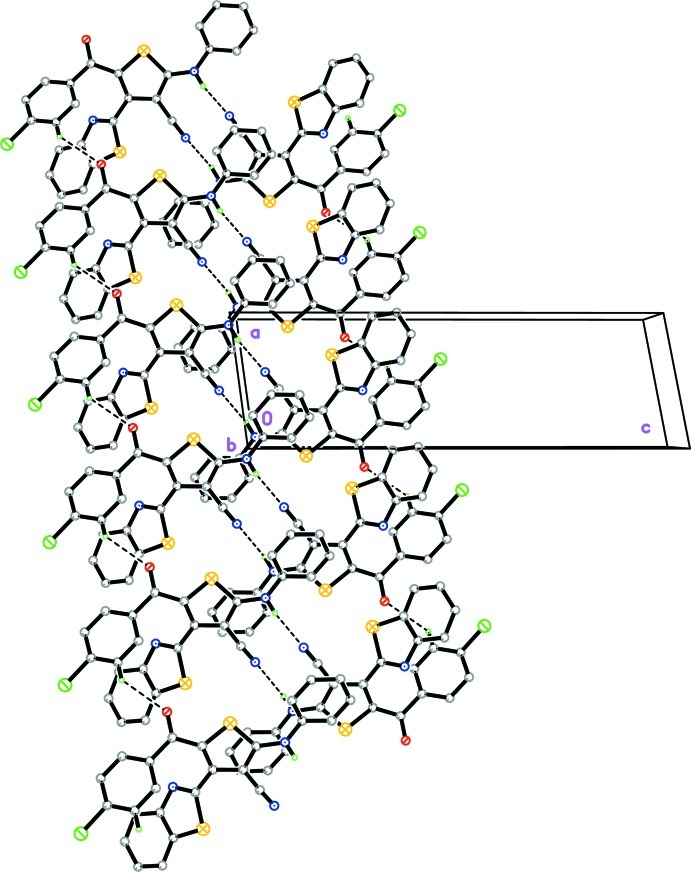
The crystal packing of the title compound. The dashed lines represent the hydrogen bonds. For clarity sake, hydrogen atoms not involved in hydrogen bonding have been omitted.

## supplementary crystallographic information

### Comment

In continuation to our interest in the chemistry of thiophenes (Fun *et al.*, 2012; Abdel-Aziz *et al.*, 2012), we report herein the crystal structure of the title compound.

The asymmetric unit of the title compound is shown in Fig. 1. The molecule consists of a thiophene ring (A) [S2/C8–C11; maximum deviation = 0.011 (1) Å at atoms S2 and C11], a chloro-substituted benzene ring (B) [C13–C18], a benzene ring (C) [C19–C24] and a benzo[*d*]thiazole ring system (D) [S1/N1/C1–C7; maximum deviation = 0.012 (1) Å at atom C7]. The dihedral angles between the mean planes of the rings are A/B = 55.72 (5)°, A/C = 13.36 (5)°, A/D = 46.77 (4)°, B/C = 67.90 (5)°, B/D = 23.99 (4)° and C/D = 60.11 (4)°. An intramolecular C24—H24A···S2 hydrogen bond generates an S(6) ring motif (Bernstein *et al.*, 1995) in the molecule.

In the crystal (Fig. 2), molecules are linked by pairs of N3—H1N3···N2 hydrogen bonds into inversion dimers and the dimers are further connected by C17—H17A···O1 hydrogen bond into tapes, running along the *a*-axis. π–π interactions are also observed with *Cg*1···*Cg*3 = 3.6116 (6) Å [symmetry code = *x*, *y*, *z*] and *Cg*2···*Cg*4 = 3.7081 (6) Å [symmetry code = -*x*, -*y*, -*z*], where *Cg*1, *Cg*2, *Cg*3 and *Cg*4 are the centroids of S1/C6/C1/N1/C7, S2/C8–C11, C13–C18 and C19–C24 rings, respectively.

### Experimental

To a stirred solution of potassium hydroxide (0.56 g, 10 mmol) in dimethylformamide (20 ml), 3-(benzo[*d*]thiazol-2-yl)-3-oxopropanenitrile (0.2 g, 1 mmol) was added. After heating at 100°C for 10 min, phenyl isothiocyanate (0.135 g, 1 mmol) was added to the resulting mixture and the heating was continued for another 10 min, then 2-chloro-1-(4-chlorophenyl)ethanone (0.189 g, 1 mmol) was added. After the addition, the reaction mixture was heated at 100°C for 15 min. The precipitated product was filtered off, washed with water and dried. Crystallization from DMF afforded the title compound. Colourless blocks were formed after slow evaporation of DMF after one month.

### Refinement

The N-bound H atom was located in a difference Fourier map and refined freely [N3—H1N3 = 0.864 (16) Å]. The remaining H atoms were positioned geometrically [C—H = 0.95 Å] and refined using a riding model with *U*_iso_(H) = 1.2*U*_eq_(C). Four outliers, (001), (026), (210) and (132) were omitted in the final refinement.

### Crystal data

**Table:**

C_25_H_14_ClN_3_OS_2_	*Z* = 2
*M**_r_* = 471.96	*F*(000) = 484
Triclinic, *P*1	*D*_x_ = 1.488 Mg m^−^^3^
Hall symbol: -P 1	Mo *K*α radiation, λ = 0.71073 Å
*a* = 6.9469 (2) Å	Cell parameters from 9847 reflections
*b* = 7.6722 (3) Å	θ = 2.9–32.6°
*c* = 20.9874 (7) Å	µ = 0.40 mm^−^^1^
α = 91.519 (1)°	*T* = 100 K
β = 97.577 (1)°	Block, colourless
γ = 107.791 (1)°	0.31 × 0.27 × 0.19 mm
*V* = 1053.15 (6) Å^3^	

### Data collection

**Table:**

Bruker APEX DUO CCD diffractometer	7637 independent reflections
Radiation source: fine-focus sealed tube	6820 reflections with *I* > 2σ(*I*)
Graphite monochromator	*R*_int_ = 0.020
φ and ω scans	θ_max_ = 32.6°, θ_min_ = 2.0°
Absorption correction: multi-scan (*SADABS*; Bruker, 2009)	*h* = −10→10
*T*_min_ = 0.884, *T*_max_ = 0.929	*k* = −11→10
27413 measured reflections	*l* = −30→31

### Refinement

**Table:**

Refinement on *F*^2^	Primary atom site location: structure-invariant direct methods
Least-squares matrix: full	Secondary atom site location: difference Fourier map
*R*[*F*^2^ > 2σ(*F*^2^)] = 0.029	Hydrogen site location: inferred from neighbouring sites
*wR*(*F*^2^) = 0.083	H atoms treated by a mixture of independent and constrained refinement
*S* = 1.04	*w* = 1/[σ^2^(*F*_o_^2^) + (0.0429*P*)^2^ + 0.3666*P*] where *P* = (*F*_o_^2^ + 2*F*_c_^2^)/3
7637 reflections	(Δ/σ)_max_ = 0.001
293 parameters	Δρ_max_ = 0.48 e Å^−^^3^
0 restraints	Δρ_min_ = −0.20 e Å^−^^3^

### Special details

**Table:**

Experimental. The crystal was placed in the cold stream of an Oxford Cryosystems Cobra open-flow nitrogen cryostat (Cosier & Glazer, 1986) operating at 100.0 (1) K.
Geometry. All e.s.d.'s (except the e.s.d. in the dihedral angle between two l.s. planes) are estimated using the full covariance matrix. The cell e.s.d.'s are taken into account individually in the estimation of e.s.d.'s in distances, angles and torsion angles; correlations between e.s.d.'s in cell parameters are only used when they are defined by crystal symmetry. An approximate (isotropic) treatment of cell e.s.d.'s is used for estimating e.s.d.'s involving l.s. planes.
Refinement. Refinement of *F*^2^ against ALL reflections. The weighted *R*-factor *wR* and goodness of fit *S* are based on *F*^2^, conventional *R*-factors *R* are based on *F*, with *F* set to zero for negative *F*^2^. The threshold expression of *F*^2^ > σ(*F*^2^) is used only for calculating *R*-factors(gt) *etc*. and is not relevant to the choice of reflections for refinement. *R*-factors based on *F*^2^ are statistically about twice as large as those based on *F*, and *R*-factors based on ALL data will be even larger.

### Fractional atomic coordinates and isotropic or equivalent isotropic displacement parameters (Å^2^)

**Table:**

	*x*	*y*	*z*	*U*_iso_*/*U*_eq_	
Cl1	0.67827 (5)	0.27031 (5)	0.480308 (13)	0.03159 (7)	
S1	0.70001 (3)	0.52966 (3)	0.221308 (11)	0.01676 (5)	
S2	−0.06551 (3)	0.19907 (3)	0.123999 (10)	0.01383 (5)	
O1	−0.14999 (11)	0.16428 (11)	0.25836 (4)	0.02145 (15)	
N1	0.43041 (12)	0.61590 (11)	0.27982 (4)	0.01415 (13)	
N2	0.56707 (13)	0.64723 (13)	0.05915 (4)	0.01993 (16)	
N3	0.07913 (11)	0.30630 (11)	0.01040 (4)	0.01366 (13)	
C1	0.61873 (14)	0.69007 (13)	0.31835 (4)	0.01479 (15)	
C2	0.64997 (16)	0.78959 (14)	0.37781 (5)	0.01997 (18)	
H2A	0.5390	0.8118	0.3949	0.024*	
C3	0.84704 (17)	0.85469 (15)	0.41095 (5)	0.0243 (2)	
H3A	0.8711	0.9229	0.4512	0.029*	
C4	1.01158 (16)	0.82196 (16)	0.38628 (5)	0.0242 (2)	
H4A	1.1449	0.8690	0.4101	0.029*	
C5	0.98442 (15)	0.72275 (15)	0.32796 (5)	0.02089 (18)	
H5A	1.0961	0.7000	0.3115	0.025*	
C6	0.78522 (14)	0.65718 (13)	0.29421 (4)	0.01608 (16)	
C7	0.45180 (13)	0.52813 (12)	0.22864 (4)	0.01309 (14)	
C8	0.28097 (13)	0.41925 (12)	0.18064 (4)	0.01252 (14)	
C9	0.28442 (13)	0.42981 (12)	0.11329 (4)	0.01245 (14)	
C10	0.10676 (13)	0.31548 (12)	0.07552 (4)	0.01227 (14)	
C11	0.10148 (13)	0.29707 (13)	0.19427 (4)	0.01379 (15)	
C12	0.03336 (14)	0.23023 (13)	0.25535 (4)	0.01508 (15)	
C13	0.19182 (14)	0.23782 (13)	0.31168 (4)	0.01467 (15)	
C14	0.16736 (15)	0.29368 (14)	0.37321 (4)	0.01761 (16)	
H14A	0.0484	0.3248	0.3793	0.021*	
C15	0.31665 (16)	0.30384 (15)	0.42550 (5)	0.02027 (18)	
H15A	0.3021	0.3438	0.4673	0.024*	
C16	0.48753 (15)	0.25455 (15)	0.41560 (5)	0.01946 (17)	
C17	0.51304 (15)	0.19385 (14)	0.35523 (5)	0.01772 (16)	
H17A	0.6296	0.1583	0.3496	0.021*	
C18	0.36368 (14)	0.18651 (13)	0.30334 (4)	0.01565 (16)	
H18A	0.3786	0.1461	0.2616	0.019*	
C19	−0.08240 (13)	0.19674 (12)	−0.03545 (4)	0.01251 (14)	
C20	−0.07628 (14)	0.24323 (13)	−0.09960 (4)	0.01489 (15)	
H20A	0.0331	0.3427	−0.1098	0.018*	
C21	−0.22951 (14)	0.14427 (14)	−0.14810 (4)	0.01668 (16)	
H21A	−0.2250	0.1767	−0.1914	0.020*	
C22	−0.39039 (14)	−0.00277 (14)	−0.13365 (4)	0.01648 (16)	
H22A	−0.4972	−0.0688	−0.1666	0.020*	
C23	−0.39207 (13)	−0.05112 (13)	−0.07028 (4)	0.01541 (15)	
H23A	−0.4994	−0.1529	−0.0604	0.018*	
C24	−0.23932 (13)	0.04676 (13)	−0.02097 (4)	0.01373 (15)	
H24A	−0.2421	0.0117	0.0221	0.016*	
C25	0.44274 (13)	0.55158 (13)	0.08486 (4)	0.01426 (15)	
H1N3	0.190 (2)	0.358 (2)	−0.0051 (7)	0.026 (4)*	

### Atomic displacement parameters (Å^2^)

**Table:**

	*U*^11^	*U*^22^	*U*^33^	*U*^12^	*U*^13^	*U*^23^
Cl1	0.03233 (14)	0.04596 (18)	0.01750 (11)	0.01807 (12)	−0.00611 (9)	−0.00167 (10)
S1	0.01263 (9)	0.02322 (12)	0.01481 (10)	0.00628 (8)	0.00207 (7)	−0.00076 (8)
S2	0.01115 (9)	0.01691 (10)	0.01187 (9)	0.00199 (7)	0.00189 (7)	0.00100 (7)
O1	0.0153 (3)	0.0294 (4)	0.0191 (3)	0.0047 (3)	0.0057 (2)	0.0047 (3)
N1	0.0140 (3)	0.0143 (3)	0.0133 (3)	0.0035 (3)	0.0015 (2)	0.0005 (3)
N2	0.0184 (4)	0.0218 (4)	0.0171 (4)	0.0019 (3)	0.0045 (3)	0.0006 (3)
N3	0.0124 (3)	0.0159 (3)	0.0111 (3)	0.0022 (3)	0.0018 (2)	0.0010 (2)
C1	0.0157 (4)	0.0142 (4)	0.0129 (3)	0.0029 (3)	0.0007 (3)	0.0008 (3)
C2	0.0236 (4)	0.0189 (4)	0.0148 (4)	0.0040 (3)	0.0007 (3)	−0.0020 (3)
C3	0.0286 (5)	0.0213 (5)	0.0166 (4)	0.0014 (4)	−0.0036 (4)	−0.0019 (3)
C4	0.0203 (4)	0.0236 (5)	0.0212 (4)	−0.0007 (4)	−0.0063 (3)	0.0037 (4)
C5	0.0142 (4)	0.0236 (5)	0.0216 (4)	0.0023 (3)	−0.0012 (3)	0.0052 (4)
C6	0.0145 (4)	0.0175 (4)	0.0145 (4)	0.0032 (3)	0.0003 (3)	0.0023 (3)
C7	0.0115 (3)	0.0148 (4)	0.0127 (3)	0.0037 (3)	0.0018 (3)	0.0011 (3)
C8	0.0119 (3)	0.0135 (4)	0.0121 (3)	0.0040 (3)	0.0018 (3)	0.0001 (3)
C9	0.0121 (3)	0.0132 (4)	0.0118 (3)	0.0034 (3)	0.0021 (3)	0.0003 (3)
C10	0.0119 (3)	0.0132 (4)	0.0121 (3)	0.0043 (3)	0.0022 (3)	0.0013 (3)
C11	0.0126 (3)	0.0165 (4)	0.0116 (3)	0.0037 (3)	0.0019 (3)	0.0010 (3)
C12	0.0161 (4)	0.0168 (4)	0.0128 (3)	0.0051 (3)	0.0038 (3)	0.0014 (3)
C13	0.0167 (4)	0.0157 (4)	0.0117 (3)	0.0044 (3)	0.0035 (3)	0.0021 (3)
C14	0.0201 (4)	0.0205 (4)	0.0139 (4)	0.0075 (3)	0.0056 (3)	0.0017 (3)
C15	0.0257 (4)	0.0237 (5)	0.0122 (4)	0.0085 (4)	0.0036 (3)	0.0001 (3)
C16	0.0219 (4)	0.0226 (5)	0.0132 (4)	0.0072 (4)	−0.0004 (3)	0.0018 (3)
C17	0.0189 (4)	0.0199 (4)	0.0156 (4)	0.0074 (3)	0.0029 (3)	0.0028 (3)
C18	0.0180 (4)	0.0171 (4)	0.0126 (3)	0.0059 (3)	0.0037 (3)	0.0019 (3)
C19	0.0119 (3)	0.0140 (4)	0.0120 (3)	0.0051 (3)	0.0009 (3)	0.0000 (3)
C20	0.0162 (4)	0.0162 (4)	0.0127 (3)	0.0057 (3)	0.0020 (3)	0.0016 (3)
C21	0.0185 (4)	0.0200 (4)	0.0123 (3)	0.0078 (3)	0.0006 (3)	0.0002 (3)
C22	0.0144 (4)	0.0200 (4)	0.0148 (4)	0.0066 (3)	−0.0005 (3)	−0.0037 (3)
C23	0.0126 (3)	0.0167 (4)	0.0167 (4)	0.0048 (3)	0.0018 (3)	−0.0021 (3)
C24	0.0128 (3)	0.0153 (4)	0.0135 (3)	0.0048 (3)	0.0025 (3)	0.0004 (3)
C25	0.0144 (3)	0.0155 (4)	0.0122 (3)	0.0042 (3)	0.0012 (3)	−0.0013 (3)

### Geometric parameters (Å, º)

**Table:**

Cl1—C16	1.7418 (10)	C9—C10	1.4017 (12)
S1—C6	1.7252 (10)	C9—C25	1.4188 (12)
S1—C7	1.7477 (9)	C11—C12	1.4754 (12)
S2—C10	1.7266 (9)	C12—C13	1.4923 (12)
S2—C11	1.7444 (9)	C13—C18	1.3963 (13)
O1—C12	1.2294 (11)	C13—C14	1.3975 (12)
N1—C7	1.3016 (11)	C14—C15	1.3900 (14)
N1—C1	1.3896 (11)	C14—H14A	0.9500
N2—C25	1.1543 (12)	C15—C16	1.3895 (14)
N3—C10	1.3517 (11)	C15—H15A	0.9500
N3—C19	1.4098 (11)	C16—C17	1.3899 (13)
N3—H1N3	0.864 (16)	C17—C18	1.3889 (13)
C1—C2	1.4016 (13)	C17—H17A	0.9500
C1—C6	1.4086 (13)	C18—H18A	0.9500
C2—C3	1.3856 (14)	C19—C24	1.3943 (12)
C2—H2A	0.9500	C19—C20	1.4046 (12)
C3—C4	1.4008 (17)	C20—C21	1.3876 (12)
C3—H3A	0.9500	C20—H20A	0.9500
C4—C5	1.3839 (16)	C21—C22	1.3967 (14)
C4—H4A	0.9500	C21—H21A	0.9500
C5—C6	1.4025 (13)	C22—C23	1.3908 (13)
C5—H5A	0.9500	C22—H22A	0.9500
C7—C8	1.4681 (12)	C23—C24	1.3940 (12)
C8—C11	1.3817 (12)	C23—H23A	0.9500
C8—C9	1.4209 (12)	C24—H24A	0.9500
			
C6—S1—C7	88.99 (4)	O1—C12—C13	121.52 (8)
C10—S2—C11	92.36 (4)	C11—C12—C13	118.41 (8)
C7—N1—C1	109.85 (8)	C18—C13—C14	119.68 (8)
C10—N3—C19	131.28 (8)	C18—C13—C12	120.18 (8)
C10—N3—H1N3	112.8 (10)	C14—C13—C12	120.13 (8)
C19—N3—H1N3	114.3 (10)	C15—C14—C13	120.15 (9)
N1—C1—C2	124.79 (9)	C15—C14—H14A	119.9
N1—C1—C6	115.30 (8)	C13—C14—H14A	119.9
C2—C1—C6	119.91 (9)	C16—C15—C14	118.89 (9)
C3—C2—C1	118.21 (10)	C16—C15—H15A	120.6
C3—C2—H2A	120.9	C14—C15—H15A	120.6
C1—C2—H2A	120.9	C15—C16—C17	122.13 (9)
C2—C3—C4	121.36 (10)	C15—C16—Cl1	119.51 (7)
C2—C3—H3A	119.3	C17—C16—Cl1	118.35 (8)
C4—C3—H3A	119.3	C18—C17—C16	118.27 (9)
C5—C4—C3	121.52 (9)	C18—C17—H17A	120.9
C5—C4—H4A	119.2	C16—C17—H17A	120.9
C3—C4—H4A	119.2	C17—C18—C13	120.84 (8)
C4—C5—C6	117.25 (10)	C17—C18—H18A	119.6
C4—C5—H5A	121.4	C13—C18—H18A	119.6
C6—C5—H5A	121.4	C24—C19—C20	119.73 (8)
C5—C6—C1	121.74 (9)	C24—C19—N3	124.30 (8)
C5—C6—S1	128.84 (8)	C20—C19—N3	115.94 (8)
C1—C6—S1	109.42 (7)	C21—C20—C19	120.18 (8)
N1—C7—C8	124.12 (8)	C21—C20—H20A	119.9
N1—C7—S1	116.43 (7)	C19—C20—H20A	119.9
C8—C7—S1	119.28 (6)	C20—C21—C22	120.36 (8)
C11—C8—C9	112.19 (8)	C20—C21—H21A	119.8
C11—C8—C7	125.47 (8)	C22—C21—H21A	119.8
C9—C8—C7	122.34 (8)	C23—C22—C21	119.06 (8)
C10—C9—C25	121.30 (8)	C23—C22—H22A	120.5
C10—C9—C8	113.63 (8)	C21—C22—H22A	120.5
C25—C9—C8	124.94 (8)	C22—C23—C24	121.31 (9)
N3—C10—C9	122.87 (8)	C22—C23—H23A	119.3
N3—C10—S2	126.75 (7)	C24—C23—H23A	119.3
C9—C10—S2	110.35 (6)	C23—C24—C19	119.31 (8)
C8—C11—C12	132.25 (8)	C23—C24—H24A	120.3
C8—C11—S2	111.44 (6)	C19—C24—H24A	120.3
C12—C11—S2	116.21 (6)	N2—C25—C9	177.03 (10)
O1—C12—C11	120.03 (8)		
			
C7—N1—C1—C2	−178.49 (9)	C9—C8—C11—C12	175.01 (9)
C7—N1—C1—C6	0.68 (11)	C7—C8—C11—C12	−5.01 (16)
N1—C1—C2—C3	179.78 (9)	C9—C8—C11—S2	−1.36 (10)
C6—C1—C2—C3	0.65 (15)	C7—C8—C11—S2	178.62 (7)
C1—C2—C3—C4	−0.30 (16)	C10—S2—C11—C8	1.75 (7)
C2—C3—C4—C5	−0.25 (17)	C10—S2—C11—C12	−175.25 (7)
C3—C4—C5—C6	0.44 (16)	C8—C11—C12—O1	158.84 (10)
C4—C5—C6—C1	−0.08 (15)	S2—C11—C12—O1	−24.93 (12)
C4—C5—C6—S1	−179.66 (8)	C8—C11—C12—C13	−23.68 (15)
N1—C1—C6—C5	−179.68 (9)	S2—C11—C12—C13	152.55 (7)
C2—C1—C6—C5	−0.46 (14)	O1—C12—C13—C18	134.78 (10)
N1—C1—C6—S1	−0.03 (10)	C11—C12—C13—C18	−42.66 (13)
C2—C1—C6—S1	179.18 (8)	O1—C12—C13—C14	−44.25 (14)
C7—S1—C6—C5	179.18 (10)	C11—C12—C13—C14	138.31 (9)
C7—S1—C6—C1	−0.44 (7)	C18—C13—C14—C15	2.09 (15)
C1—N1—C7—C8	174.18 (8)	C12—C13—C14—C15	−178.87 (9)
C1—N1—C7—S1	−1.05 (10)	C13—C14—C15—C16	−1.14 (15)
C6—S1—C7—N1	0.90 (8)	C14—C15—C16—C17	−0.60 (16)
C6—S1—C7—C8	−174.57 (7)	C14—C15—C16—Cl1	178.87 (8)
N1—C7—C8—C11	−44.54 (14)	C15—C16—C17—C18	1.34 (16)
S1—C7—C8—C11	130.56 (8)	Cl1—C16—C17—C18	−178.14 (8)
N1—C7—C8—C9	135.44 (10)	C16—C17—C18—C13	−0.35 (14)
S1—C7—C8—C9	−49.46 (11)	C14—C13—C18—C17	−1.34 (14)
C11—C8—C9—C10	0.11 (11)	C12—C13—C18—C17	179.63 (9)
C7—C8—C9—C10	−179.88 (8)	C10—N3—C19—C24	−11.48 (15)
C11—C8—C9—C25	175.87 (9)	C10—N3—C19—C20	170.28 (9)
C7—C8—C9—C25	−4.11 (14)	C24—C19—C20—C21	2.20 (13)
C19—N3—C10—C9	177.25 (9)	N3—C19—C20—C21	−179.48 (8)
C19—N3—C10—S2	−4.70 (14)	C19—C20—C21—C22	−0.31 (14)
C25—C9—C10—N3	3.59 (14)	C20—C21—C22—C23	−1.52 (14)
C8—C9—C10—N3	179.53 (8)	C21—C22—C23—C24	1.51 (14)
C25—C9—C10—S2	−174.74 (7)	C22—C23—C24—C19	0.36 (13)
C8—C9—C10—S2	1.20 (10)	C20—C19—C24—C23	−2.20 (13)
C11—S2—C10—N3	−179.90 (8)	N3—C19—C24—C23	179.61 (8)
C11—S2—C10—C9	−1.66 (7)		

### Hydrogen-bond geometry (Å, º)

**Table:**

*D*—H···*A*	*D*—H	H···*A*	*D*···*A*	*D*—H···*A*
N3—H1*N*3···N2^i^	0.862 (15)	2.163 (15)	2.9558 (12)	152.7 (13)
C17—H17*A*···O1^ii^	0.95	2.60	3.3496 (14)	136
C24—H24*A*···S2	0.95	2.49	3.1719 (9)	128

Symmetry codes: (i) −*x*+1, −*y*+1, −*z*; (ii) *x*+1, *y*, *z*.
